# A computer vision system for deep learning-based detection of patient mobilization activities in the ICU

**DOI:** 10.1038/s41746-019-0087-z

**Published:** 2019-03-01

**Authors:** Serena Yeung, Francesca Rinaldo, Jeffrey Jopling, Bingbin Liu, Rishab Mehra, N. Lance Downing, Michelle Guo, Gabriel M. Bianconi, Alexandre Alahi, Julia Lee, Brandi Campbell, Kayla Deru, William Beninati, Li Fei-Fei, Arnold Milstein

**Affiliations:** 1 Department of Computer Science, Stanford University, 353 Serra Mall, Stanford, CA 94305 USA; 2 Clinical Excellence Research Center, Stanford University School of Medicine, 75 Alta Rd, Stanford, CA 94305 USA; 3 Division of General Surgery, Department of Surgery, Stanford University School of Medicine, 300 Pasteur Drive, Rm H3680, Stanford, CA 94305 USA; 4 Department of Medicine, Center for Biomedical Informatics Research, Stanford University School of Medicine, 1265 Welch Road, Stanford, CA 94305 USA; 5 School of Architecture, Civil and Environmental Engineering, École Polytechnique Fédérale de Lausanne, 1015 Lausanne, Switzerland; 6 Department of Critical Care Medicine, LDS Hospital, Intermountain Healthcare, 8th Avenue, C St. E, Salt Lake City, UT 84143 USA

**Keywords:** Health services, Computer science

## Abstract

Early and frequent patient mobilization substantially mitigates risk for post-intensive care syndrome and long-term functional impairment. We developed and tested computer vision algorithms to detect patient mobilization activities occurring in an adult ICU. Mobility activities were defined as moving the patient into and out of bed, and moving the patient into and out of a chair. A data set of privacy-safe-depth-video images was collected in the Intermountain LDS Hospital ICU, comprising 563 instances of mobility activities and 98,801 total frames of video data from seven wall-mounted depth sensors. In all, 67% of the mobility activity instances were used to train algorithms to detect mobility activity occurrence and duration, and the number of healthcare personnel involved in each activity. The remaining 33% of the mobility instances were used for algorithm evaluation. The algorithm for detecting mobility activities attained a mean specificity of 89.2% and sensitivity of 87.2% over the four activities; the algorithm for quantifying the number of personnel involved attained a mean accuracy of 68.8%.

## Introduction

Survivors of prolonged, high-intensity care frequently suffer from post-intensive care syndrome, characterized by long-term cognitive and physical impairment leading to a significant decline in functional status.^1–3^ Mobilization of critically ill patients can shorten time to weaning from mechanical ventilation, reduce delirium, and prevent muscle wasting and dysfunction (ICU-acquired weakness).^4–7^ This is significant, as these are preventable harms that impact overall survival, the ability to independently perform activities of daily living, and health-related quality of life.^8–10^ Although early studies indicate benefit of mobility interventions in select patient groups,^4,11,12^ much more-detailed studies are needed to determine how variations in the type, frequency, and duration of mobilization activities impact outcomes for this diverse patient population.^13,14^ Unfortunately, the scope of such studies is currently limited, as implementation of early mobility protocols requires overcoming substantial organizational and cultural barriers,^15^ and success has historically been difficult to measure.

Current practices for monitoring patient mobility include direct human observation^16^ and mining of the electronic health record (EHR) for documentation of mobility events.^17^ These methods are time and labor intensive, prone to inaccurate documentation, and involve a notable time lag between patient care and reporting. Computer vision technology (CVT) offers an alternative approach by passively capturing data from the clinical environment, with application of machine-learning algorithms to detect and quantify patient and staff activities automatically.^18^ Indeed, there has been increasing interest in using CVT to perform activity recognition and improve patient care in hospitals.^19^ For instance, computer vision algorithms have been developed to perform automated recognition of hand hygiene events in hospital corridors^20^ and trauma resuscitation events in the emergency department.^21,22^ CVT has also been applied in the operating room, where algorithms recognize patient care tasks (such as moving the patient onto the operating table), steps and tools in a surgical procedure, and even the surgeon’s level of operative skill.^23,24^ Finally and most relevant to our study, Ma et al.^25^ used CVT to determine a numeric mobility level for patients in a single ICU room. We build off of this work by using depth sensor-based CVT to collect data from seven individual adult ICU rooms and develop machine-learning algorithms to temporally detect patients’ bedside activities and the healthcare personnel involved.

## Results

### Algorithm performance for detection of mobility activities

**Fig. 1:**
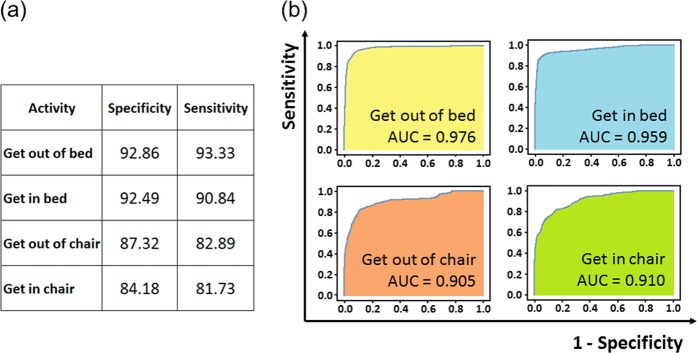
Algorithm performance for detecting the occurrence of mobility activities. **a** Per-class specificity and sensitivity, evaluated at the frame-level. **b** Per-class receiver operating characteristic curves (ROC). These ROC curves demonstrate the trade-off between sensitivity (the true positive rate) and 1-specificity (the false-positive rate), as the detection thresholds are varied. The area under the ROC curve (AUC) is an aggregate measure of detection performance, and indicates the probability that the model will rank a positive example more highly than a negative example (a model whose predictions are 100% correct will have an AUC of 1.0)

The algorithm for detection of mobility activity occurrence achieved a mean sensitivity and specificity of 87.2% and 89.2%, respectively, and a mean area under the curve of 0.938, over all four activities when evaluating prediction at the level of individual frames of video data (frame-level prediction). Per-activity breakdown and receiver operating characteristic curves are shown in Fig. 1. Frame-level predictions were merged to determine the duration of the mobility activities detected by the algorithm. The mean duration for all mobility activities predicted by the algorithm was 7.6 s (standard deviation 12.6 s, min 0.4 s, max 146.5 s, for durations of individual mobility activities, see Supplementary Data 1). For comparison, the mean duration of all activities as based on the manually reviewed, annotated data (ground truth) was 9.0 s (standard deviation 12.9 s, min 0.5 s, max 123.9 s, see Supplementary Table 1 for a comparison of algorithm-predicted and ground truth activity durations). Activities were both correctly classified and had predicted durations within + /− 15% of the ground truth standard duration for 58.1% of activities; within + /− 25 for 68.7% of activities; and within + /− 50% for 82.0% of activities.

### Algorithm performance for detection of healthcare personnel

**Fig. 2:**
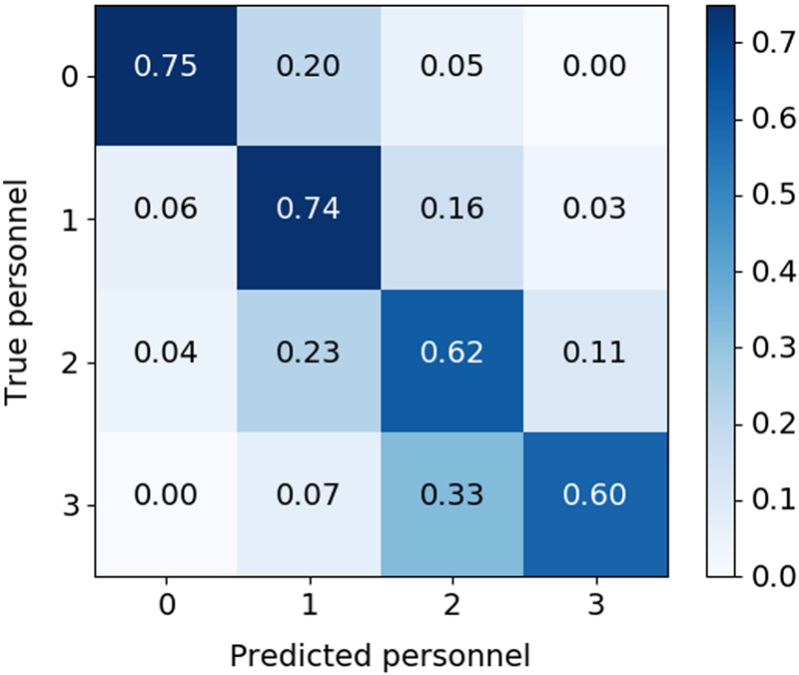
Algorithm performance for quantifying the number of healthcare personnel involved in mobility activities. A confusion matrix is shown for true number of healthcare personnel assisting with mobility activity instances (numbered 0–3), vs. the number of personnel detected by the algorithm. When a patient mobilizes alone, the number of detected healthcare personnel is reported as 0. When a patient mobilizes with one healthcare personnel assisting, this is reported as 1, etc. Values are normalized across each row (true number of personnel)

The algorithm for quantifying the number of healthcare personnel involved in each activity achieved a mean accuracy of 68.8%. A confusion matrix for distribution of true vs. predicted personnel during mobility activities is shown in Fig. 2. The confusion matrix demonstrates that when a patient mobilizes alone, the algorithm correctly detects this (0 predicted personnel) 75% of the time; when there is a single healthcare worker present, the algorithm correctly detects this (1 predicted personnel) 74% of the time. Detection accuracies for 2 and 3 healthcare personnel were 62% and 60%, respectively. The algorithm correctly detects 2 or more-predicted personnel (as opposed to 0 or 1) 78% of the time (see Supplementary Figure 1).

**Fig. 3:**
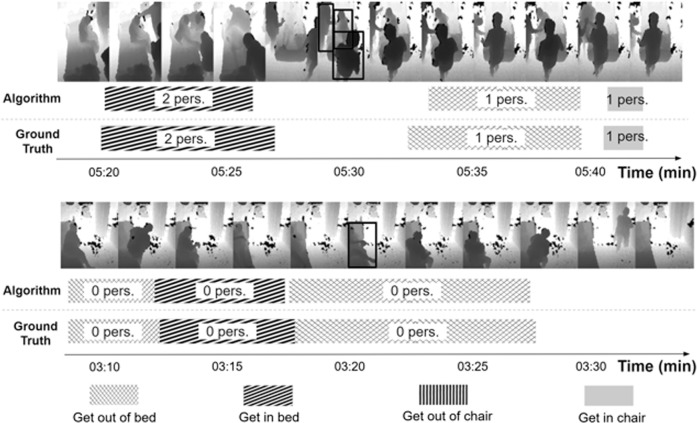
Timelines of mobility activity occurrence and healthcare personnel involvement. Two timelines from condensed periods of time in patient rooms are shown. In each timeline, sampled depth image frames from the period are shown. Spatial bounding boxes of person detections are also overlaid (shown only in the center frame for ease of visualization). The temporal extents and number of healthcare personnel (abbreviated “pers.”) involved in each activity (taking into account that one person detected corresponds to the patient) are indicated on the timeline. Human-annotated ground truth is shown for comparison

Figure 3 shows qualitative examples of the algorithm outputs. Sampled depth image frames from two (condensed) periods of time inside patient rooms are shown. Beneath these, timelines are shown indicating detected activities and their temporal occurrence, duration, and number of healthcare personnel involved. Comparison with the ground truth standard is also shown.

## Discussion

We show that computer vision algorithms can accurately detect patient mobility activities, their duration, and the number of personnel that complete them. Although our study builds on the work of Ma et al.,^25^ whose algorithm calculates a numeric mobility score for ICU patients, our algorithms enable more detailed study of how specific types of mobility events and variation in their frequency and duration will impact clinical outcomes. This aspect of our work is clinically significant, as there is currently great variation in protocols for early mobilization of critically ill patients, which limits the generalizability of study findings.^26^ Overall, our method represents a clinically useful tool for quantifying patient mobility practices in real time, and provides proof-of-concept that more comprehensive mobility data may be collected using CVT. Ultimately, it is this level of granularity that will allow clinicians to hone in on the most effective mobility practices in order to refine and standardize mobility protocols. In addition to aiding in refinement of mobility protocols, our algorithms may also be used to provide insights into how they may be most effectively implemented. Limitations on multidisciplinary staffing and workload are cited as major barriers to implementation of patient mobility protocols.^15^ Thus, our CVT-based method to quantify the staffing and time required to complete patient care activities promises to enhance our understanding of barriers or facilitating factors that contribute to adoption of best practices. Moreover, this approach could be applied to other healthcare activities, and may be used to augment time-directed activity-based costing^27^ and other methods to define the resources required for delivering optimal care.

The performance of our algorithm for detection of mobility activities differs between the types of activities. We do not anticipate that current differences in detecting the selected mobility activities will limit the ability to utilize the algorithm for downstream studies, as these levels of sensitivity and specificity should be sufficient to detect the broader clinical trends. Interestingly, these differences shed light on the relative difficulty of detecting some activities as compared with others. For example, the activities getting into/out of a chair may be more difficult to detect because these events tend to be shorter in duration (with ground truth mean durations of 3.1 s and 2.7 s, respectively). Similarly, the algorithm for detection of healthcare personnel assisting with mobility activities reached a mean accuracy of 68.8%. Notably, most of the errors occur in distinguishing between two and three personnel, which may be attributed to occlusion in the sensor viewpoint when more than two people are present to assist the patient. We hope to address this in future studies by incorporating additional sensor viewpoints into the data stream. Overall, it is important to note that these levels of accuracy are a strong starting point for clinical deployment of the algorithms in this study. As we continue to collect and expose the algorithms to additional data, allowing them to see more examples of these mobility activities in a variety of different physical environments, we expect that these differences in performance will minimize over time.

Previous methods for studying mobility practices for critically ill patients have relied on direct human observation or retrospective review of documented mobility events in the EHR. The primary disadvantage of these methods is that they are time and labor intensive, and provide only sparse temporal coverage (as human observers cannot practically collect data 24 h a day). These data collection methods are therefore difficult to scale to enable large clinical studies. In fact, a recent systematic review suggests that many clinical correlation studies examining the impact of early mobility protocols do not reach statistical significance precisely owing to a lack of sufficient, quality data.^14^ In contrast to these methods, CVT collects data 24 h a day, eliminates the need for direct observation, and reduces susceptibility to error from variations in rater reliability or recall. Our computer vision algorithms, now that they have been developed, can be deployed continuously and at very little additional labor cost to detect the real-time occurrence of mobility activities at the scale needed to enable useful downstream clinical studies. Furthermore, we demonstrate that CVT can facilitate additional descriptive analyses of these activities beyond just occurrence, such as their duration and the number of healthcare workers assisting with the activity. Nevertheless, challenges to our approach remain. To develop these algorithms, substantial cognitive labor was needed to manually annotate data and obtain sufficient training examples for temporally sparse activities such as those examined. We were able to partially mitigate this challenge by developing a web-based application for nurses to flag the approximate time occurrences of witnessed patient mobility activities. This streamlined the review and manual annotation of flagged mobility events by research assistants, and allowed us to generate a large curated data set of mobility activity examples. Despite these multiple layers of annotation, human labeling of data for algorithm development remains laborious and is still susceptible to error (for inter-rater reliability calculations, see Methods). However, an advantage of using this CVT-based method is that once the algorithms have been developed, they may also be deployed to new environments with relatively little additional annotation of data. Known as “fine-tuning”, labeling of a limited number of additional training examples in a new setting can allow the algorithms to quickly achieve a high level of performance in that setting. Our algorithms were trained and evaluated using data from seven patient rooms in a single ICU setting. Thus, we do not yet have empirical evidence for how our approach would perform in a significantly different environment. Nonetheless, the strong performance of these algorithms in the current setting with seven rooms indicates promise for effective generalization to other environments. In addition, we anticipate that once the algorithms are exposed to data from multiple institutions, they will learn institution-level generalization such that the need for fine-tuning will eventually be eliminated.

Overall, we describe an automated approach to detect intended patient care activities and propose that the method could be used to generate critical insights to promote effective and efficient early mobility protocols for critically ill patients.

## Methods

### Study participants

The study was conducted in the adult ICU of Intermountain LDS Hospital (Salt Lake City, Utah). Participants included patients admitted to rooms equipped with computer vision depth sensors between August and October 2017, as well as staff entering these rooms. The purpose of this study was to develop and validate computer vision algorithms to detect the occurrence of patient mobility activities, as well as other descriptive attributes of mobility activities such as their duration and the number of personnel assisting. As such, we did not access patient clinical data or quantify the number of patients monitored, as this information was not necessary to validate algorithmic performance. The study protocol was approved by the Intermountain Healthcare Institutional Review Board. Informed consent was waived because the protocol posed no more than minimal risk to participants.

### Data collection and annotation

Depth sensors capture 3D volumetric images of humans and objects based on their distance from the sensor, thereby providing visual information while preserving privacy. Sensors were mounted directly facing the bed in seven individual patient rooms, and image data were collected 24 h a day during the study period (2 months). Supplementary Figure 2 shows a floor plan for the Intermountain LDS Hospital ICU, including the location of each sensor and the relative configuration of each room in the study.

To create a curated data set of mobility event occurrences for model training and evaluation, data were manually reviewed and annotated by trained research assistants for four separate activities related to patient mobilization: patient getting into and out of bed, and patient getting into and out of chair. The number of personnel assisting with each mobility activity was also annotated. Owing to the temporal sparsity of patient mobility activities (making it difficult to find and annotate occurrences in long stretches of recorded data), a web-based application was developed to allow nursing staff to flag the approximate time occurrences of the patient mobility activities they witnessed, providing research assistants with a time stamp in the data for focused retrospective review. The use of time stamps to coarsely indicate the occurrence of mobility events enabled our research assistants to retrospectively examine only the periods of data flagged by nursing staff to identify and label mobility activities, avoiding manual review of thousands of hours of data. Three trained research assistants reviewed these sampled periods of data to provide precise temporal annotations, with each occurrence of a mobility activity being reviewed by one research assistant. To assess consistency of the manual review across the different research assistants, a subset of the data was annotated by all three of the research assistants. Frame-level inter-rater reliability of annotations on this subset was 0.894 using Fleiss’s kappa.^28^

### Training and test data sets

A total of 563 mobility events were annotated and included in the final, curated data set, comprising 154 instances of patient getting out of bed, 182 of getting into bed, 112 of getting out of chair, and 115 of getting into chair. The final data set included 98,801 frames of data, totaling 5.7 h. From the collected data set, 67% of the mobility activity instances and surrounding frames were randomly used for training, and 33% for testing. As such, 379 instances of patient mobility activities were used for training, and the remaining 184 instances of patient mobility activities were used for testing. The test data set included 48 instances of patient getting out of bed, 64 of patient getting into bed, 32 of patient getting out of chair, and 40 of patient getting into chair.

### Augmentation of training data set

An augmentation data set was used during the training of the neural network for temporal detection of mobility activities and their duration. In order to improve algorithm performance, additional data comprising simulations of the targeted mobility activities was used to augment the training set during model development. These simulations were conducted to provide scripted instances of mobility activities over a short period of time, making them less labor intensive to manually annotate as compared to non-simulation activities that occur infrequently over long stretches of time. This data was collected during clinician-led mobility activity simulations in two of the seven patient rooms equipped with computer vision sensors in the LDS Hospital ICU, as well as in a dedicated patient simulation room at Stanford University. In total, data collected during simulations added 318 additional occurrences of mobility activities, totaling 41,353 frames of additional training data. This additional data included 97 instances of patient getting out of bed, 93 of patient getting into bed, 59 of patient getting out of chair, and 69 of patient getting into chair. Supplementary Figure 3 shows how simulation data were incorporated into the training data set. The simulation data were used only for improving training of the model (by providing an additional 318 training examples) and not for evaluation of algorithm accuracy, such that the evaluation remains based only on patient data. We chose not to include any simulation data in the test data set to evaluate the neural network because we felt that it would be a less-direct measure of how the algorithm would perform on data from a real-world, patient care environment.

Supplementary Table 2 shows the performance statistics for the algorithm with and without the addition of the simulation data to the training data set. Obtaining training data through simulation was a useful technique to enhance the neural network’s performance in a time-efficient manner, and improved the mean sensitivity and specificity on the evaluation data set from 82.93 and 84.44% to 87.20 and 89.20%, respectively. Adding the simulation data provided more examples for all activity classes and increased the exposure to variability in the training data. A comparison of the AUC (an aggregate measure of classification performance) for each activity class shows the improvement obtained with the addition of the simulation data to the training set (Supplementary Figure 4).

### Model for detection of mobility activities and their duration

The algorithm for temporal detection of the mobility activities and their duration was a multi-label recurrent convolutional neural network model.^29^ We used an 18-layer ResNet convolutional neural network^30^ pre-trained on the large-scale ImageNet^31^ and fine-tuned on our data set to initially extract informative visual features from every frame of data. We subsequently used a two-layer bidirectional long short-term memory recurrent network to reason over temporal structure in consecutive 64-frame sequences of these features. An ensemble of six such models was used to produce the final detection output.

### Model for detection of healthcare personnel

The algorithm for quantifying the number of personnel involved in each mobility activity was based on the YOLOv2^32^ convolutional neural network architecture for object detection. The YOLOv2 convolutional neural network was trained to predict the spatial locations of people in each image frame of data using annotated bounding boxes of the spatial locations of people in 1379 frames of patient data. This trained person-detector was evaluated to achieve a spatial average precision of 0.66 compared with human annotation. After applying the person-detector to the image data, post-processing was used to smooth detections over time. The maximum number of detected people over the duration of a mobility activity (taking into account that one person is the patient) was used to quantify the number of healthcare personnel involved in each activity. In the data set, 7% of activities had a true number of 0 healthcare personnel involved, 51% had one healthcare personnel, 32% had two healthcare personnel, and 10% had three healthcare personnel.

### Evaluation of algorithm performance

Evaluation of the algorithms’ accuracy was assessed by comparing the manual annotations of the data set (known as the ground truth standard) with the predictions made by the algorithms. Sensitivity, specificity, and receiver operating characteristic calculations were performed using Python 3.6 (Python Software Foundation, https://www.python.org/).

### Code availability

Full code is available from the authors upon reasonable request.

## Supplementary information

Supplementary Material

## Data Availability

The data that support the findings of this study are available from Intermountain Healthcare, but restrictions apply to the availability of these data, which were used under license for the current study, and so are not publicly available. Data are, however, available from the authors upon reasonable request and with permission of Intermountain Healthcare.

## Supplementary information

**Supplementary information** accompanies the paper on the *npj Digital Medicine* website (10.1038/s41746-019-0087-z).
