# Supplementary material for: A computer vision system for deep learning-based detection of patient mobilization activities in the ICU
Source: NPJ Digit Med. 2019 Mar 1;2:11. doi: 10.1038/s41746-019-0087-z (PMC6550251; doi:10.1038/s41746-019-0087-z)
Supplement: Supplementary file 1 — Supplementary Material [file 41746_2019_87_MOESM1_ESM.pdf]

Supplementary Data 1: Duration of Mobility Activities

Mean duration, standard deviation (SD), and minimum and maximum durations for individual mobility activities as detected by the algorithm were as follows: patient getting into bed 9.5 s (SD 11.2 s, min 0.4 s, max 101.9 s), patient getting out of bed 11.4 s (SD 18.7 s, min 0.4 s, max 146.5 s), patient getting into chair 4.0 s (SD 5.4 s, min 0.4 s, max 39.9 s); patient getting out of chair 3.5 s (SD 5.0 s, min 0.4 s, max 38.1 s). For comparison, the mean duration, standard deviation (SD), and minimum and maximum durations for individual mobility activities as based on the manually reviewed, annotated data (ground truth) were as follows: patient getting into bed 10.4 s (SD 8.5 s, min 0.6 s, max 82.0 s), patient getting out of bed 13.5 s (SD 18.4 s, min 0.6 s, 123.9 s), patient getting into chair 3.1 s (SD 3.5 s, min 0.5 s, max 20.1 s), patient getting out of chair 2.7 s (SD 4.6 s, min 0.5 s, max 28.1 s). A side-by-side comparison of the algorithm-predicted and ground truth activity durations is provided in Supplementary Table 1.

Supplementary Tables and Figures:

Activity	Ground Truth Durations (s) Mean (SD, min, max)	Algorithm Predicted Durations (s) Mean (SD, min, max)
**All activities**	9.0 s (SD 12.9, min 0.5, max 123.9)	7.6 s (SD 12.6, min 0.4, max 146.5)
**Getting into bed**	10.4 s (SD 8.5, min 0.6, max 82.0)	9.5 s (SD 11.2, min 0.4, max 101.9)
**Getting out of bed**	13.5 s (SD 18.4, min 0.6, max 123.9)	11.4 s (SD 18.7, min 0.4, max 146.5)
**Getting into chair**	3.1 s (SD 3.5, min 0.5, max 20.1)	4.0 s (SD 5.4, min 0.4, max 39.9)
**Getting out of chair**	2.7 s (SD 4.6, min 0.5, max 28.1)	3.5 s (SD 5.0, min 0.4, max 38.1)

Supplementary Table 1: Comparison of ground truth and algorithm-predicted activity durations. The mean, standard deviation (SD), minimum (min) and maximum (max) durations are shown for the manually reviewed, human-annotated data (ground truth) versus the durations predicted by the algorithm.

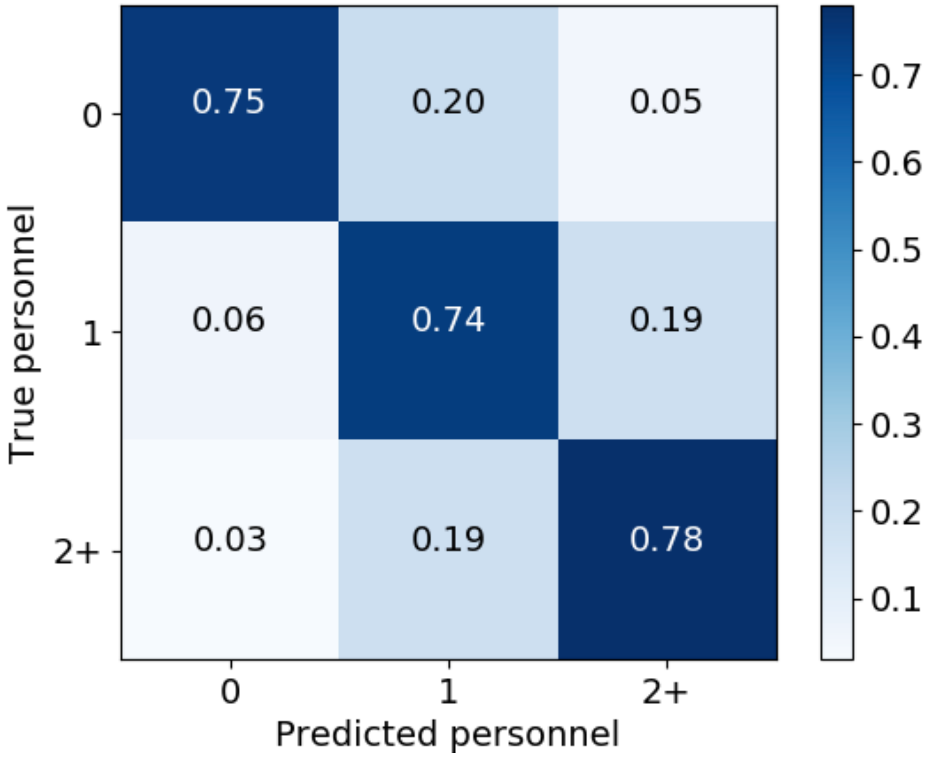

Supplementary Figure 1: Confusion matrix for algorithm performance when quantifying 0, 1 or 2+ healthcare personnel assisting with mobility activities. A confusion matrix is shown for true number of healthcare personnel assisting with mobility activity instances (numbered 0, 1 or 2+), vs. the number of personnel detected by the algorithm. When a patient mobilizes alone, the number of detected healthcare personnel is reported as 0. When a patient mobilizes with one healthcare personnel assisting, this is reported as 1. When a patient mobilizes with 2 or more healthcare personnel, this is reported as 2+. Values are normalized across each row (true number of personnel).

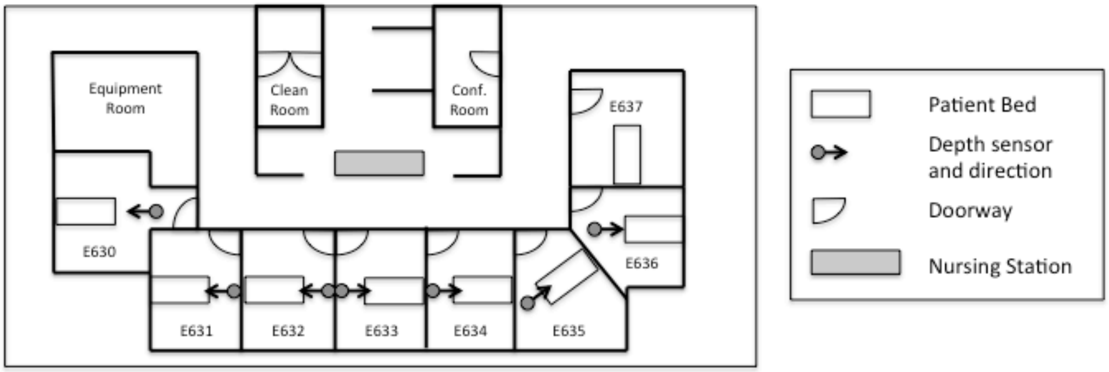

Supplementary Figure 2: Layout of patient rooms and sensor placement in the Intermountain LDS Hospital ICU. Seven patient rooms were equipped with depth sensors. Sensors were installed directly facing the bed in each room.

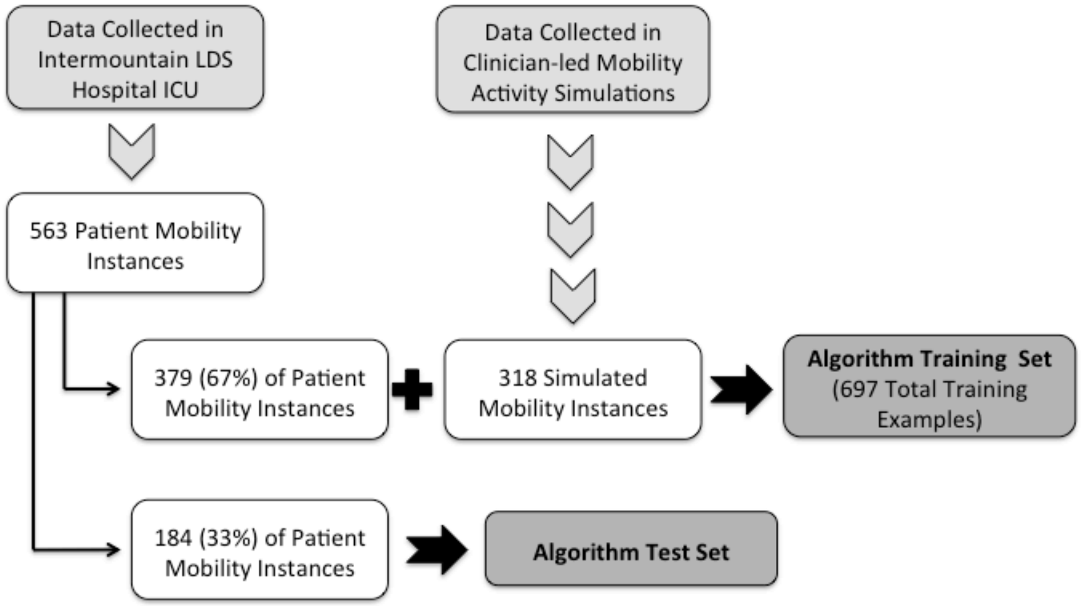

Supplementary Figure 3: Training and test datasets used in model development for detection of mobility activities and their duration. During the training phase, the algorithm was exposed to a total of 697 mobility activity examples, including 384 from depth video data collected in the LDS Hospital ICU, and 318 collected during clinician-led mobility activity simulations. The algorithm was tested on a held-out set of 184 patient mobility activity instances collected in the LDS Hospital ICU.

Activity	Algorithm Performance, Trained with Patient Data Alone		Algorithm Performance, Trained with Patient Data and Simulation Data	
	Sensitivity	Specificity	Sensitivity	Specificity
**All activities (mean)**	82.93	84.44	87.20	89.20
**Get out of bed**	93.29	85.48	93.33	92.86
**Get in bed**	84.90	90.79	90.84	92.49
**Get out of chair**	77.30	80.41	82.89	87.32
**Get in chair**	76.23	81.06	81.73	84.18

Supplementary Table 2: **Performance comparison to quantify the effect of training with simulation data for the algorithm detecting mobility activities.** Algorithm performance using 2 distinct training datasets is shown. On the left-hand side of the table, algorithm performance using a training dataset comprised of patient data alone (379 total mobility activity occurrences) is shown. On the right-hand side, algorithm performance is demonstrated when using a training dataset that contains both patient mobility instances and an additional 318 instances from simulation data (for a total of 697 training examples).

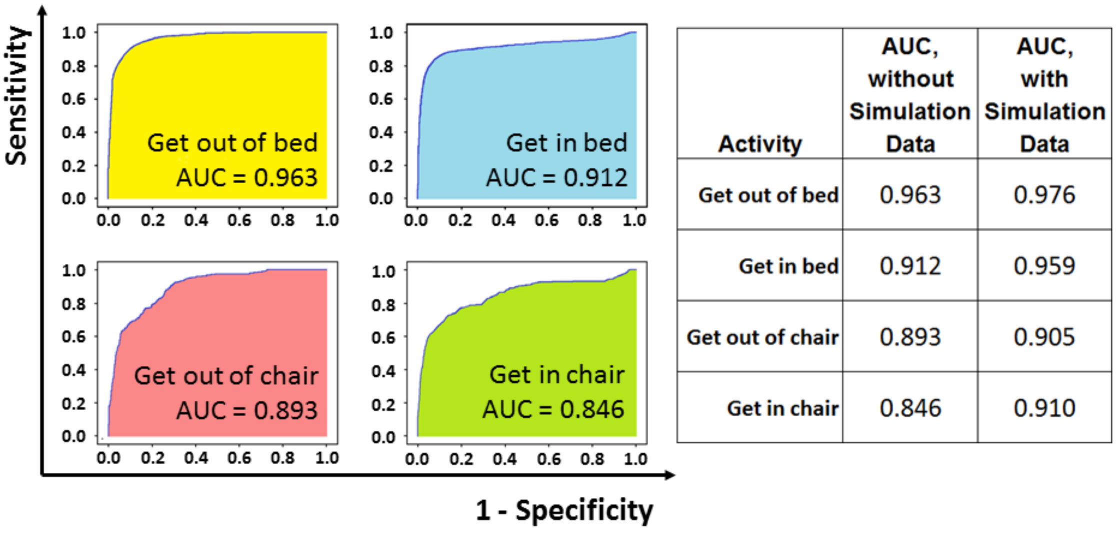

(a)

Activity	AUC, without Simulation Data	AUC, with Simulation Data
Get out of bed	0.963	0.976
Get in bed	0.912	0.959
Get out of chair	0.893	0.905
Get in chair	0.846	0.910

(b)

Supplementary Figure 4: **Receiver operating characteristic curves for algorithm performance when training with patient data alone**. (a) Per-class receiver operating characteristic (ROC) curves are shown. The area under the ROC curve (AUC) for each class is also shown. (b) The AUC is an aggregate measure of performance and can be compared to the AUC values for algorithm performance when trained on a dataset containing both patient data and simulation data.
